# Familial Bell's Palsy: A Case Report and Literature Review

**DOI:** 10.1155/2012/674981

**Published:** 2012-08-05

**Authors:** Mark Kubik, Liliana Robles, Doris Kung

**Affiliations:** Department of Neurology, Baylor College of Medicine, Houston, TX 77030, USA

## Abstract

*Objective*. To describe a unique case of familial Bell's palsy and summarize the current literature regarding possible hereditary influences. *Design*. Case report. *Main Outcome Measures*. Clinical exam, CSF analysis, and family history provided per the patient. *Results*. We report the case of a 58-year-old female who presented with recurrent and bilateral episodes of facial palsy. The patient underwent multiple CSF investigations to rule out a possible infectious and rheumatologic etiology that were all negative. Further questioning revealed she was one of seven family members with a history of unilateral facial nerve paralysis. *Conclusion*. The sheer number of similar case studies to date suggests that familial clustering of Bell's palsy is a real, noncoincidental phenomenon. Our case represents a unique and perplexing example of one such family. Familial Bell's palsy may represent an autoimmune disease secondary to inherited HLA alloantigens or a structural predisposition to disease based on the dimensions of the facial canal.

## 1. Introduction

Bell's palsy, or idiopathic facial paralysis (IFP), accounts for approximately 60–80% of lower motor neuronal facial palsies [1] and has an incidence of 20–30 cases per 100,000 people per year [2]. Incidence is highest in the 15–45 year age group and increases in pregnancy and diabetes [3]. In the vast majority of cases, it is self-limited, nonprogressive, and spontaneously remitting with a very small minority of patients left with residual neurologic dysfunction [4]. A positive family history has been estimated to be present in approximately 4–14% of cases [2]. 

## 2. Case Presentation

**Figure 1:**
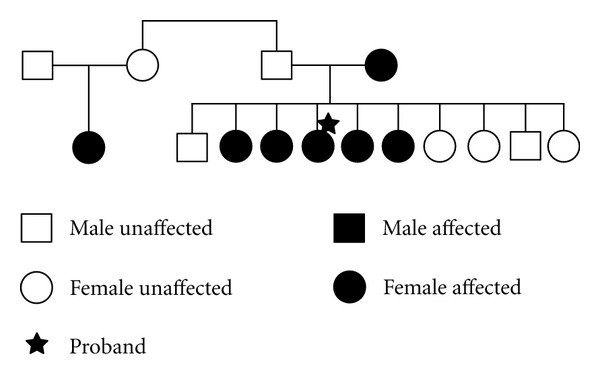
Pedigree of patient's family with 7 affected individuals across 2 generations.

A 58-year-old Hispanic woman with no significant past medical history presented to a county hospital in 2007 due to the sudden onset of peripheral left facial weakness preceded by an upper respiratory tract infection. She received acyclovir and a course of prednisone with a partial response documented 4 weeks later. Three months after the initial episode, she returned to the emergency department due to relapse of the left peripheral facial weakness. A second cycle of acyclovir and prednisone was prescribed, and she underwent brain magnetic resonance imaging (MRI) and Lyme serology testing. Brain MRI without contrast was unremarkable and her Lyme IgG/IgM antibodies were equivocal with negative a Western Blot (WB). In early 2010, the patient was seen yet again in an outpatient clinic for a 2-day history of left facial palsy; this time she declined steroids. Concerns were raised regarding the equivocal serology for Lyme and recurrence of facial palsy, and the patient was empirically treated with doxycycline for 2 weeks. A WB for Lyme disease was repeated and returned positive. She was evaluated by the infectious disease service and provided a second course of antibiotics. In June 2011, the patient presented to the emergency department with sudden onset of right facial weakness and was admitted for further workup. Results were significant for negative WB for Lyme disease, cerebrospinal fluid (CSF), analysis revealed WBC 2, RBC 1, glucose 95 mg/dL, and protein 31 mg/dL. CSF polymerase chain reaction (PCR) for Lyme, varicella zoster virus (VZV), and herpes simplex virus (HSV) was negative. The IgG synthesis rate was 3.6, which was slightly elevated but there were no oligoclonal bands (OGB). Rheumatologic panel included positive antinuclear antibodies (ANA) 1 : 80 speckled, double stranded ANA was negative, and antiribonucleoprotein was borderline positive. Upon reviewing her clinical history, the patient mentioned having 6 relatives with histories consistent with peripheral Bell's palsy, including her mother, one cousin, and 4 of her sisters. One of the patient's sisters also had recurrent episodes of facial nerve paralysis. The familial pedigee is displayed in Figure 1.

## 3. Discussion

Since David McCormicks's landmark study published in Lancet in 1972, the most commonly cited mechanism for IFP involves reactivation of latent herpes simplex virus in the seventh nerve ganglion [5]. However, there still exists tremendous controversy regarding its etiology and risk factors. Additional postulates have implicated anatomic, autoimmune, vascular, enzymatic deficiency, and immune dysregulation as potential causal factors.

While a firm hereditary basis for IFP has yet to be uncovered, familial clustering has long been noted in the literature. Since the first reported case series of familial Bell*ʼ*s palsy by Neumann in 1887, several case studies and series have been published describing patient subsets with extremely high familial incidences. In one of the more extensive case series analyses, Wilbrant and Blumhagen describe a population of 230 individuals with idiopathic peripheral seventh nerve palsy, 6% of which had family history. Interestingly enough, one family had a total of 29 cases of Bell's palsy and a very high degree of consanguinity in the family [6]. Estimates regarding the incidence of familial Bell's palsy have found that a positive family history exists in approximately 4–14% of cases [7, 8]. Among the familial cases identified, there has been no difference found in the severity of the palsy, residual deficits, recovery time, response to medical therapy, or recurrence relative to nonfamilial cases [4]. The most commonly cited mode of inheritance is autosomal dominant with variable penetrance [8].

Numerous studies have attempted to provide objective evidence of a genetic basis for IFP. Much of the attention thus far has centered on the human leukocyte antigen (HLA) system, which has strong objective associations with a variety of autoimmune diseases. One Mexican study of 92 patients with IFP found a significant decrease in the HLA class 2 DR antigen and acutely decreased levels of CD3/CD4 T cells at the onset of facial paralysis, suggesting the possibility that an HLA-DR linked “resistance gene” may exist [9]. In those individuals with decreased DR antigen and IFP, a familial antecedent was noted in a remarkable 46% of cases. Additional studies have corroborated this association with the HLA DR locus, specifically implicating antigens DR2, DR4, DRW6, DRW7 in disease pathogenesis [10]. Shibahara [11], in a study of 93 patients with Bell's palsy, also found significantly higher levels of HLA antigens Bw67 and Cw7 in IFP patients relative to the controls [12]. In addition, they found that those positive for Bw67 and Bw7 also tended to have specific clinical courses characterized by bilateral or recurrent episodes. The presence of Cw7 antigen was also found to be associated with a higher incidence of persistent facial paralysis at followup.

Ultimately, these studies have yielded a convoluted and mixed understanding of the role of HLA antigens in IFP. We feel these studies provide evidence of, if nothing else, a state of immune dysregulation that often accompanies IFP. Specifically, there seems to be a repeated theme of HLA-DR antigen positivity and the subsequent development of IFP. Rather than a simple inflammatory response to reactivation of HSV in the nerve ganglion, IFP may very well represent a secondary autoimmune disease induced by infection. Many studies have postulated that familial IFP is, thus, an autoimmune disease with genetic predisposition. While an interesting theory, it has little experimental backing. All the current evidence exists in the form of retrospective genetic analyses, all of which have produced possible associations with various antigens and nothing more. Additional, more extensive, rigorous retrospective analyses are needed to further characterize this possible genetic component to IFP. 

Among mechanisms posed for IFP, a structural predisposition has long been mentioned as a potential contributor. Knowledge of the pathophysiology of this disease, with the exception of the proven association with HSV, has been historically been limited to edema and swelling of the facial nerve. This has been objectively documented both on MRI contrast enhancement and direct observation during decompression operations. Contrast enhancement, interestingly, has been often noted bilaterally and not simply restricted to the side of facial nerve dysfunction [13]. Seropositivity to HSV is relatively common, and yet IFP remains a relatively rare disease in comparison. Could the development of clinically evident BP be dependent on the presence of a structural narrowing or constriction along the course of the facial nerve? Such an anatomic anomaly could determine whether a patient experiences clinically manifest Bell's palsy or subclinical facial nerve edema. Viral-induced facial nerve swelling in a spatially restricted canal could cause a transient paralysis similar to other traumatic neuropraxias.

Though a paucity of evidence exists with regard to this question, the few imaging-based studies to date have found evidence that seems to support the validity of this hypothesis. One retrospective computed-tomography-based study of 25 patients with IFP found a statistically significant asymmetry in diameter of the facial canal at the meatal foramen between clinically involved and uninvolved sides [13]. This study also noted evidence of bilateral contrast enhancement of the facial nerve in several of the subjects. One additional study found a higher incidence of narrowing at the stylomastoid foramen among IFP patients [14]. That the side of Bell's palsy correlates with the presence of an anatomic narrowing suggests a very real and intuitive association of IFP and spatial features of the facial canal. To date, however, there have been no studies detailing anatomic variations in familial cases. It is certainly plausible that inherited abnormalities or congenital defects in embryonic facial canal formation create a strictly physical predisposition to later disease. Such a mechanical predisposition could explain the high incidence of familial clustering seen in the literature.

## 4. Conclusion

Bell's palsy remains a complex and mysterious entity from a pathophysiologic perspective. Seropositivity to HSV is a clearly established risk factor in the development of IFP. However, the clear discord between the incidence of HSV seropositivity and the incidence of IFP suggests the progression of disease from infection with HSV to facial nerve paralysis is a very multifactorial process dependent on several host factors. 

The sheer number of case studies to date suggests that familial clustering of IFP is a real, noncoincidental phenomenon. Our case represents a unique and perplexing example of one such family. Our patient had recurrent episodes of paralysis that occurred in the absence of an obvious precipitant. Her workup revealed no evidence of hyperglycemia to suggest diabetic neuropathy, unremarkable imaging, and negative serologic studies for infection. Her facial palsy was thus felt to be due to an underlying genetic predisposition, though we were unable to obtain HLA testing of this individual. Whether this family's history represents common exposure to similar environmental influences or a discrete genetic or anatomic predisposition is ultimately unknown, but the current body of evidence seems to strongly support a possible hereditary basis.
